# EARLY-LIFE WAR EXPOSURE AND LATER-LIFE FRAILTY AMONG OLDER ADULTS IN VIETNAM: DOES WAR HASTEN AGING?

**DOI:** 10.1093/geroni/igad104.0444

**Published:** 2023-12-21

**Authors:** Zachary Zimmer, Kim Korinek, Yvette Young, Bussarawan Teerawichitchainan, Tran Khanh Toan

**Affiliations:** Mount Saint Vincent University, Halifax, Nova Scotia, Canada; University of Utah, Salt Lake City, Utah, United States; University of Utah, Salt Lake City, Utah, United States; National University of Singapore, Singapore, Singapore; Hanoi Medical University, Dong Da, Ha Noi, Vietnam

## Abstract

We aimed to assess the nature and degree of association between exposure to potentially traumatic wartime experiences in early life and later-life frailty. The Vietnam Health and Aging Study included war survivors in Vietnam, age 60+. Latent class analysis (LCA) is used to construct classes exposed to similar numbers and types of wartime experiences. Frailty is measured using a deficit accumulation approach that approximates biological aging. LCA yields 9 unique wartime exposure classes, ranging from extreme exposure to non-exposed. Higher frailty levels among those with heavy/severe exposures certain combinations of experiences, including intense bombing, witnessing death firsthand, having experienced sleep disruptions during wartime, and having feared for one’s life during war. The difference in frailty-associated aging between the most and least affected individuals is more than 18 years. War trauma hastens aging and warrants greater attention toward long-term implications of war on health among vast post-conflict populations across the globe.

